# Supplementary material for: Transcription activation by a sliding clamp
Source: Nat Commun. 2021 Feb 18;12:1131. doi: 10.1038/s41467-021-21392-0 (PMC7892883; doi:10.1038/s41467-021-21392-0)
Supplement: Supplementary file 1 — Supplementary Information [file 41467_2021_21392_MOESM1_ESM.pdf]

Transcription activation by a sliding clamp

Shi et al.

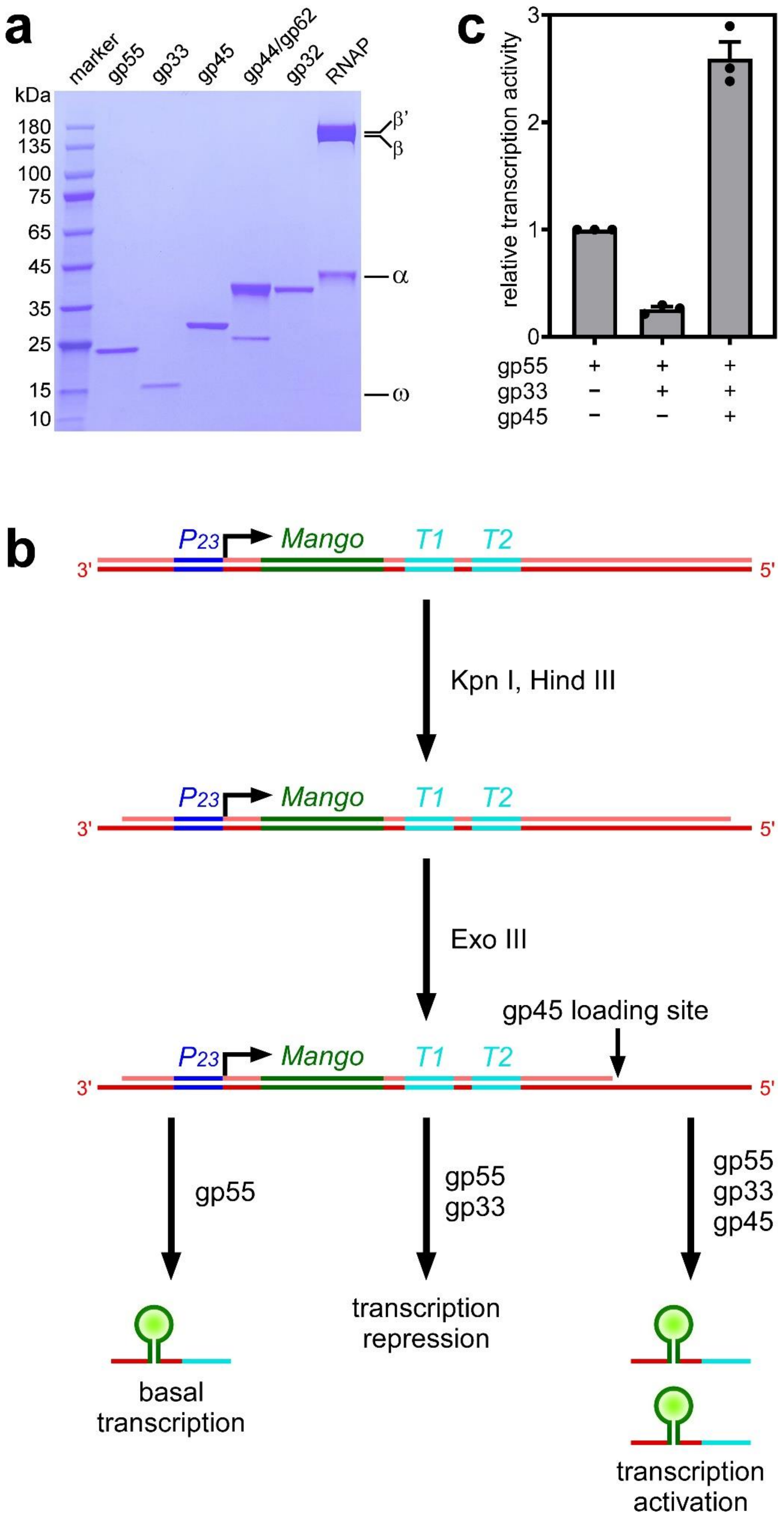

Supplementary Figure 1. Reconstitution of gp55-dependent transcription and gp45-

dependent transcription activation in vitro.

a SDS-PAGE of purified proteins. Experiments were repeated independently three times with similar results.

b The principle of Mango III transcription assay.

c Gp55-dependent transcription is activated by gp33 and gp45. Error bars represent mean ± SEM out of n = 3 experiments.

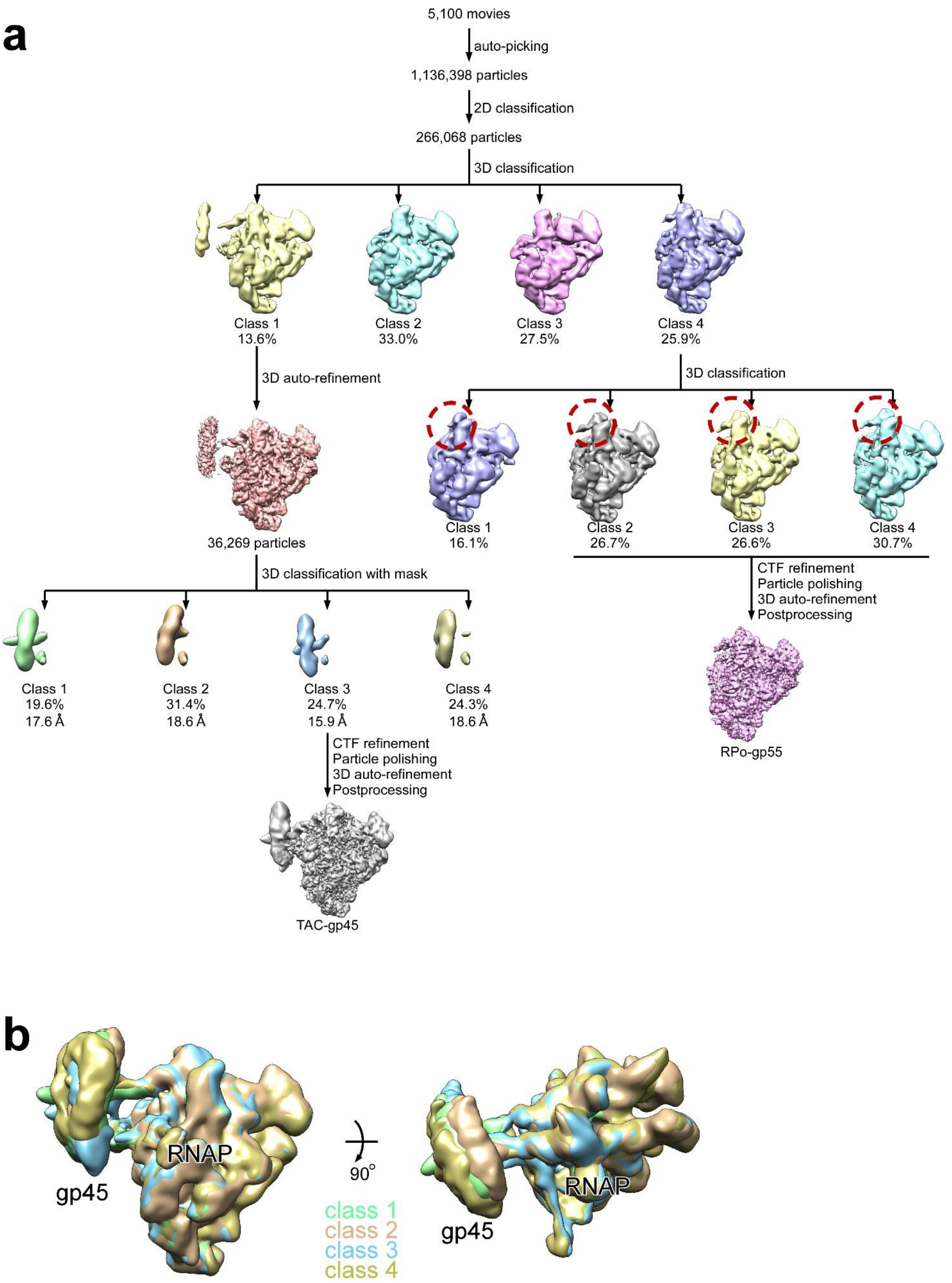

Supplementary Figure 2. Data processing pipeline.

a Initial 3D classification results in a reconstruction with densities for gp45 (TAC-gp45) and a

reconstruction without densities for gp45 (RPo-gp55). For RPo-gp55, further 3D classification with 4 classes is performed. The density map of the upstream dsDNA of class 1 (in the dashed circle) is weak compared with the other three classes. Therefore, only classes 2-4 are combined for refinement. For TAC-gp45, masked 3D classification leads to four classes with slightly different conformations of gp45 relative to RNAP, which is consistent with the model that gp45 is tethered to RNAP through flexible linkers. Class 3 is analyzed in detail because its resolution is the highest among the four classes. Predicted local resolution of gp45 is indicated below each class.

b Superposition of four classes of TAC-gp45 shows slightly different conformations of gp45 relative to RNAP.

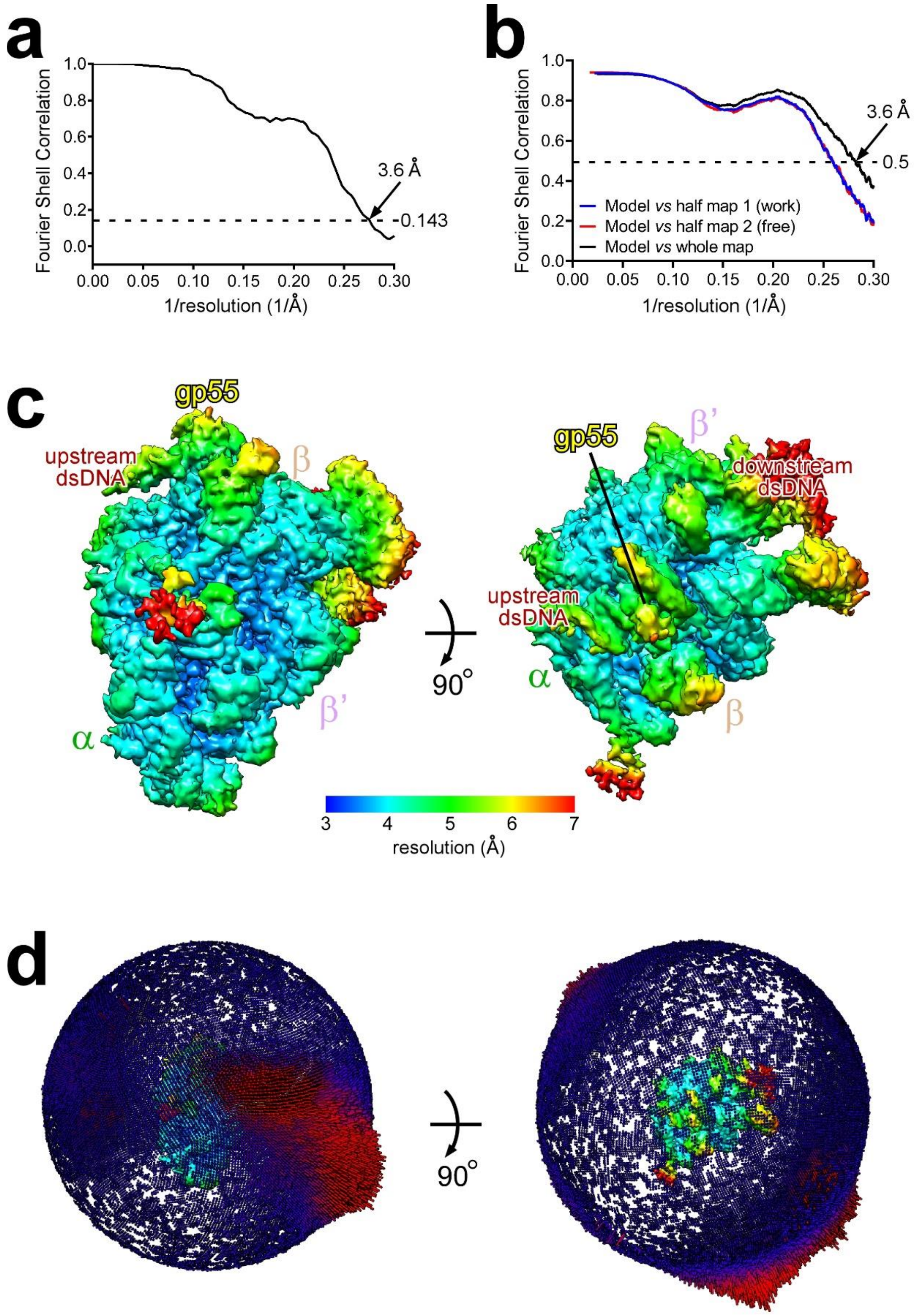

Supplementary Figure 3. Data validation for RPo-gp55.

a Gold-standard FSC. The gold-standard FSC was calculated by comparing the two independently determined half-maps from RELION. The dashed line represents the 0.143 FSC cutoff.

b FSC calculated between the model and the half map used for refinement (work), the other half map (free), and the full map.

c Cryo-EM density map colored by local resolution. Local resolution calculation was performed using blocres [1]. View orientations as in Fig. 1b.

d Angular distribution of particle projections. View orientations as in Fig. 1b.

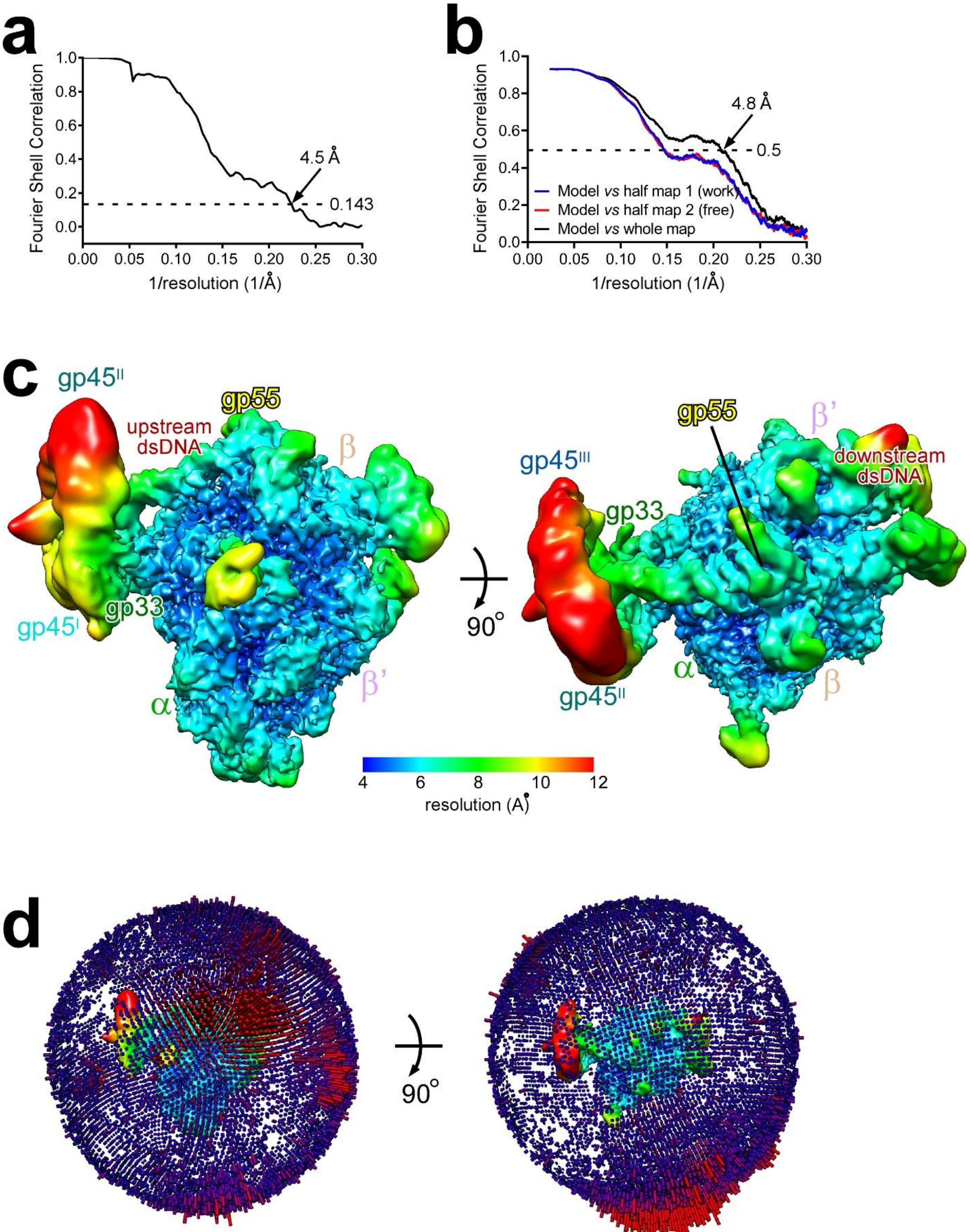

Supplementary Figure 4. Data validation of TAC-gp45.

a Gold-standard FSC. The gold-standard FSC was calculated by comparing the two independently determined half-maps from RELION. The dashed line represents the 0.143 FSC cutoff.

b FSC calculated between the model and the half map used for refinement (work), the other half map (free), and the full map.

c Cryo-EM density map colored by local resolution. Local resolution calculation was performed using blocres. View orientations as in Fig. 1c.

d Angular distribution of particle projections. View orientations as in Fig. 1c.

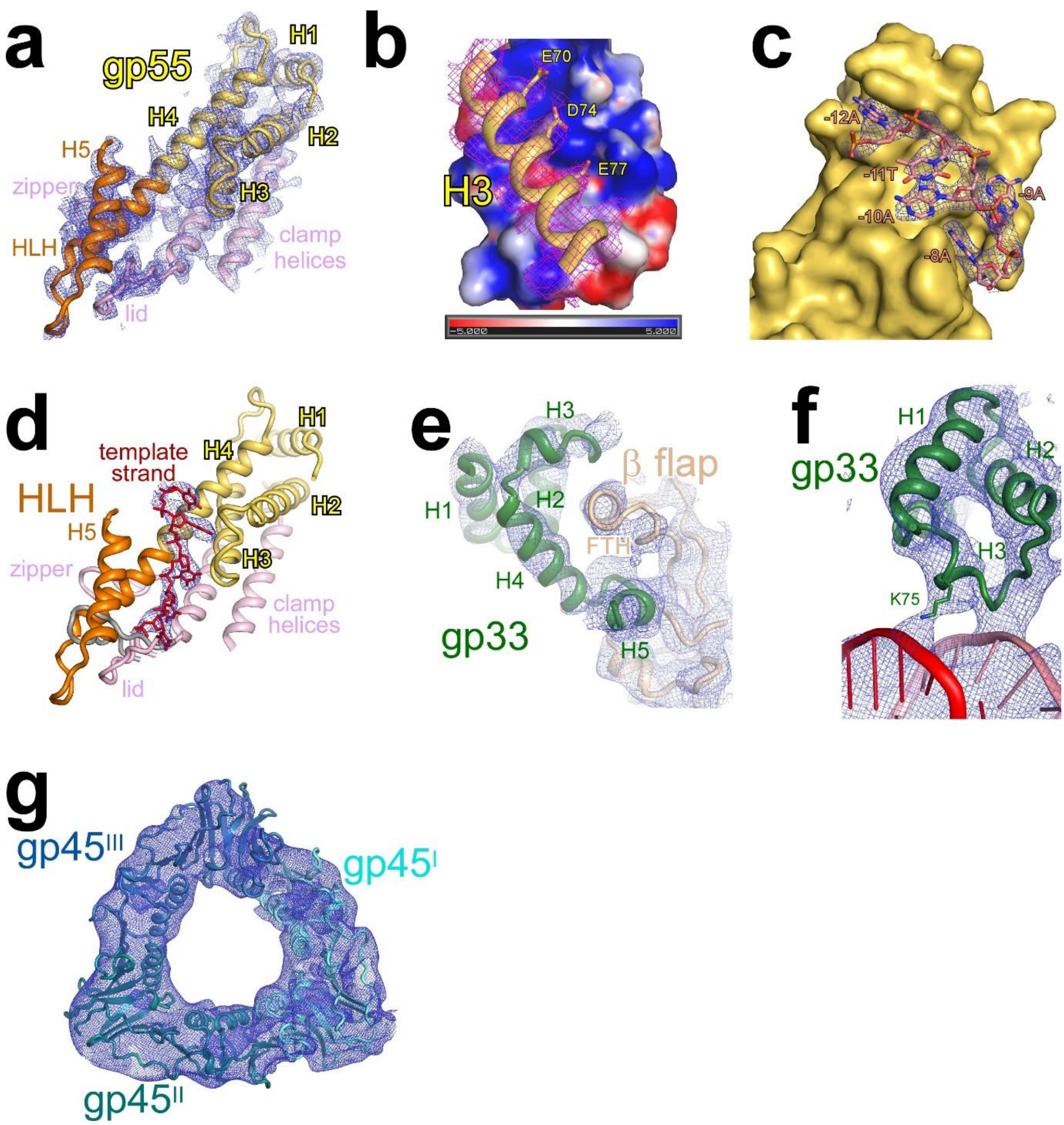

Supplementary Figure 5. Representative cryo-EM densities and superimposed models.

a Cryo-EM density map (blue mesh) and the superimposed model of gp55, the clamp helices, the zipper, and the lid. View orientation and colors as in Fig. 2b.

b Cryo-EM density map (magenta mesh) and the superimposed model of gp55 H3. View orientation and colors as in Fig. 2d.

c Cryo-EM density map (blue mesh) and the superimposed model of nontemplate-strand ssDNA. View orientation and colors as in Fig. 3a.

d Cryo-EM density map (blue mesh) and the superimposed model of template-strand ssDNA. View orientation and colors as in Fig. 4a.

e Cryo-EM density map (blue mesh) and the superimposed model of gp33 and the β flap. View orientation and colors as in Fig. 5a.

f Cryo-EM density map (blue mesh) and the superimposed model of gp33 and the upstream dsDNA. View orientation and colors as in Fig. 5c.

g Cryo-EM density map (blue mesh) and the superimposed model of gp45. View orientation and colors as in Fig. 6a.

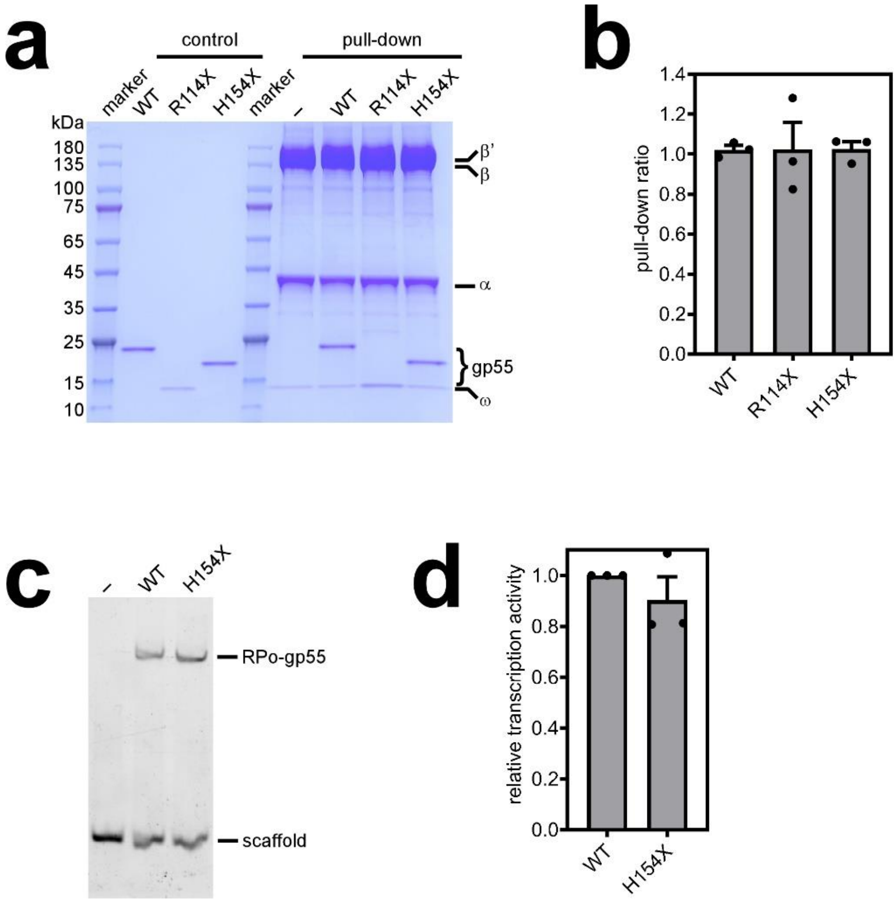

Supplementary Figure 6. The C-terminal region truncated gp55 binds RNAP, forms RPo-gp55, and initiates basal transcription as effectively as full-length gp55.

a Pull-down experiments show that truncation of the C-terminal region and the HLH motif doesn't affect RNAP-gp55 holoenzyme formation. In order to truncate the C-terminal region, the codon for residue H154 is mutated to a stop codon (H154X). In order to truncate the HLH motif and the C-terminal region, the codon for residue R114 is mutated to a stop codon (R114X). Experiments were repeated independently three times with similar results.

b Quantification of the pull-down experiments. Error bars represent mean ± SEM out of n = 3 experiments.

c Electrophoretic mobility shift assays show that truncation of the C-terminal region doesn't affect RPo-gp55 formation. Experiments were repeated independently three times with similar results.

d Mango III transcription assays show that truncation of the C-terminal region doesn't affect gp55-dependent transcription. Error bars represent mean ± SEM out of n = 3 experiments.

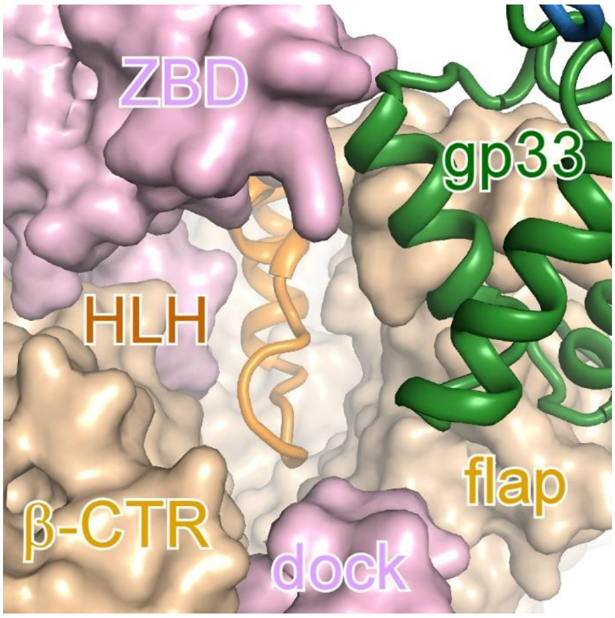

Supplementary Figure 7. The HLH motif of gp55 blocks the RNA exit channel, while gp33 does not.

Dark green, gp33; wheat, β subunit C-terminal region (β-CTR) and flap; light pink, zinc binding domain (ZBD) and dock; orange, helix-loop-helix (HLH) motif.

Supplementary Table 1. Cryo-EM data collection and refinement statistics.

	RPo-gp55	TAC-gp45
**Data collection and processing**		
Microscope	Titan Krios	Titan Krios
Voltage (kv)	300	300
Detector	K2 summit	K2 summit
Electron exposure (e/Å^2)	59	59
Defocus range (μm)	1.5-2.5	1.5-2.5
Data collection mode	Counting	Counting
Physical pixel size (Å/pixel)	1.307	1.307
Symmetry imposed	C1	C1
Initial particle images	1,136,398	1,136,398
Final particle images	57,776	8,981
Map resolution (Å)[a]	3.6	4.5
**Refinement**		
Map sharpening B-factor (Å^2)	-55	-94
Root-mean-square deviation		
Bond lengths (Å)	0.007	0.008
Bond angles (°)	0.768	0.922
Molprobity statistics		
Clashscore	17	33
Rotamer outliers (%)	0.11	0.55
Cβ outliers (%)	0.00	0.00
Ramachandran plot		
Favored (%)	88.15	84.35
Outliers (%)	0.06	0.02

[a]Gold-standard FSC 0.143 cutoff criteria.

Supplementary Table 2. Sequence alignment of SCBMs.

Protein	Sequence
Gp33	106 N T **L D F L L** 112
Gp55	175 P S **L D F L Y** E 182
T4 DNA ligase	229 E G **L D F L F** D 236

Supplementary Table 3. Primers used in this study.

Primer name	sequence (5' to 3')
forward primer to amplify nucleic-aicd scaffold	atgtgctgcaaggcgattaagttgg
reverse primer to amplify nucleic-aicd scaffold	ggctcgtatgttgtgtggaattgtg
forward primer to generate gp55 mutant protein N13A	caaaatacaactacgtgaacgccaaggagctgctgcaagcc
reverse primer to generate gp55 mutant protein N13A	ggcttgcagcagctccttggcgttcacgtagttgtattttg
forward primer to generate gp55 mutant protein L54A	gctgattgcggaaggtgcgagcaagcgcttcaac
reverse primer to generate gp55 mutant protein L54A	gttgaagcgcttgctcgcaccttccgcaatcagc
forward primer to generate gp55 mutant protein R57A	ggaaggtctgagcaaggccttcaacttcagcggc
reverse primer to generate gp55 mutant protein R57A	gccgctgaagttgaaggccttgctcagaccttcc
forward primer to generate gp55 mutant protein F86A	aacgccgacgagacgaagtacaagaacccgcac
reverse primer to generate gp55 mutant protein F86A	ttcgtctcgtcggcgttgtgcagacctttgatgctcg
forward primer to generate gp55 mutant protein H95A	cgaagtacaagaacccggccgcgtacatcacccaag
reverse primer to generate gp55 mutant protein H95A	cttgggtgatgtacgcggccgggttcttgtacttcg
forward primer to generate gp55 mutant protein Y97A	caagaacccgcacgcggccatcacccaagcgtg
reverse primer to generate gp55 mutant protein Y97A	cacgcttgggtgatggccgcgtgcgggttcttg
forward primer to generate gp55 mutant protein T99A	cgcacgcgtacatcgcccaagcgtgcttcaac
reverse primer to generate gp55 mutant protein T99A	gttgaagcacgcttgggcgatgtacgcgtgcg
forward primer to generate gp55 mutant protein R114A	cagcgcatcaaaaaggaggccaaggaggtggcgaagaag
reverse primer to generate gp55 mutant protein R114A	cttcttcgccacctccttggcctcctttttgatgcgctg
forward primer to generate gp55 mutant protein R114X	gttcagcgcatcaaaaaggagtagaaggaggtggcgaagaagtac
reverse primer to generate gp55 mutant protein R114X	gtacttcttcgccacctccttctactcctttttgatgcgctgaac
forward primer to generate gp55 mutant protein H154X	tatctacgacaagatgacgtagtacgaggaaagcacctacc
reverse primer to generate gp55 mutant protein H154X	ggtaggtgctttcctcgtactacgtcatcttgtcgtagata
forward primer to generate gp33 mutant protein K75A	ctattcctgaaactcaatttgctgcatttattccttcgggtataattg
reverse primer to generate gp33 mutant protein K75A	caattatacccgaaggaataaatgcagcaaattgagtttcaggaatag

Supplementary References

1. Cardone, G., Heymann, J.B. and Steven, A.C. One number does not fit all: mapping local variations in resolution in cryo-EM reconstructions. *J. Struct. Biol.* **184**, 226-236 (2013).
