# Supplementary material for: Transcription activation by a sliding clamp
Source: Nat Commun. 2021 Feb 18;12:1131. doi: 10.1038/s41467-021-21392-0 (PMC7892883; doi:10.1038/s41467-021-21392-0)
Supplement: Supplementary file 3 — Reporting Summary [file 41467_2021_21392_MOESM3_ESM.pdf]

nature research

Corresponding author(s): Yu Feng

Last updated by author(s): Jan 7, 2021

Reporting Summary

Nature Research wishes to improve the reproducibility of the work that we publish. This form provides structure for consistency and transparency in reporting. For further information on Nature Research policies, see our Editorial Policies and the Editorial Policy Checklist.

Statistics

For all statistical analyses, confirm that the following items are present in the figure legend, table legend, main text, or Methods section.

n/a	Confirmed	
☐	☒	The exact sample size (*n*) for each experimental group/condition, given as a discrete number and unit of measurement
☐	☒	A statement on whether measurements were taken from distinct samples or whether the same sample was measured repeatedly
☒	☐	The statistical test(s) used AND whether they are one- or two-sided<br>*Only common tests should be described solely by name; describe more complex techniques in the Methods section.*
☒	☐	A description of all covariates tested
☒	☐	A description of any assumptions or corrections, such as tests of normality and adjustment for multiple comparisons
☐	☒	A full description of the statistical parameters including central tendency (e.g. means) or other basic estimates (e.g. regression coefficient) AND variation (e.g. standard deviation) or associated estimates of uncertainty (e.g. confidence intervals)
☒	☐	For null hypothesis testing, the test statistic (e.g. *F*, *t*, *r*) with confidence intervals, effect sizes, degrees of freedom and *P* value noted<br>*Give P values as exact values whenever suitable.*
☒	☐	For Bayesian analysis, information on the choice of priors and Markov chain Monte Carlo settings
☒	☐	For hierarchical and complex designs, identification of the appropriate level for tests and full reporting of outcomes
☒	☐	Estimates of effect sizes (e.g. Cohen's *d*, Pearson's *r*), indicating how they were calculated

Our web collection on statistics for biologists contains articles on many of the points above.

Software and code

Policy information about availability of computer code

Data collection	Images were recorded with Serial EM 3.8 (Mastronarde, 2005).
Data analysis	The following computer softwares were used for data analysis:<br>WinCoot 0.9.2 (Emsley et al., 2010)<br>Phenix 1.16 (Adams et al., 2010)<br>MotionCor2 1.1.0 (Zheng et al., 2017)<br>CTFFIND 4.1 (Rohou and Grigorieff, 2015)<br>RELION 3.0 (Scheres, 2012)<br>Chimera 1.11.2 (Pettersen et al., 2004)<br>Blocres 2.0.7 (Cardone et al., 2013)<br>Image J 1.52a

For manuscripts utilizing custom algorithms or software that are central to the research but not yet described in published literature, software must be made available to editors and reviewers. We strongly encourage code deposition in a community repository (e.g. GitHub). See the Nature Research guidelines for submitting code & software for further information.

Data

Policy information about availability of data

All manuscripts must include a data availability statement. This statement should provide the following information, where applicable:

- Accession codes, unique identifiers, or web links for publicly available datasets
- A list of figures that have associated raw data
- A description of any restrictions on data availability

The accession codes for the cryo-EM density maps reported in this paper are Electron Microscopy Data Bank: EMD-30604 [https://www.emdataresource.org/EMD-30604] (RPo-gp55) and EMD-30605 [https://www.emdataresource.org/EMD-30605] (TAC-gp45). The accession codes for the atomic coordinates reported in this paper are Protein Data Bank: 7D7C [https://doi.org/10.2210/pdb7D7C/pdb] (RPo-gp55) and 7D7D [https://doi.org/10.2210/pdb7D7D/pdb] (TAC-gp45). The accession code for the cryo-EM density map used in this study is EMD-8585 [https://www.emdataresource.org/EMD-8585]. The accession codes for the atomic coordinates used in this study are PDB 6CA0 [https://doi.org/10.2210/pdb6CA0/pdb], PDB 3TBI [https://doi.org/10.2210/pdb3TBI/pdb], and PDB 6DRT [https://doi.org/10.2210/pdb6DRT/pdb]. Source data are provided with this paper. Other data are available from the corresponding author upon reasonable request.

Field-specific reporting

Please select the one below that is the best fit for your research. If you are not sure, read the appropriate sections before making your selection.

☒ Life sciences ☐ Behavioural & social sciences ☐ Ecological, evolutionary & environmental sciences

For a reference copy of the document with all sections, see nature.com/documents/nr-reporting-summary-flat.pdf

Life sciences study design

All studies must disclose on these points even when the disclosure is negative.

Sample size	Not relevant (The current work doesn't involve population study).
Data exclusions	None.
Replication	Biochemical experiments were performed in triplicate. Results were consistent.
Randomization	Not relevant (The current work doesn't involve population study).
Blinding	Not relevant (The current study doesn't involve experimental subjects with opinions).

Reporting for specific materials, systems and methods

We require information from authors about some types of materials, experimental systems and methods used in many studies. Here, indicate whether each material, system or method listed is relevant to your study. If you are not sure if a list item applies to your research, read the appropriate section before selecting a response.

Materials & experimental systems

n/a	Involved in the study	
☒	☐	Antibodies
☒	☐	Eukaryotic cell lines
☒	☐	Palaeontology and archaeology
☒	☐	Animals and other organisms
☒	☐	Human research participants
☒	☐	Clinical data
☒	☐	Dual use research of concern

Methods

n/a	Involved in the study	
☒	☐	ChIP-seq
☒	☐	Flow cytometry
☒	☐	MRI-based neuroimaging
